# Association of foveal avascular zone with the metamorphopsia in epiretinal membrane

**DOI:** 10.1038/s41598-020-74190-x

**Published:** 2020-10-13

**Authors:** Hideki Shiihara, Hiroto Terasaki, Shozo Sonoda, Naoko Kakiuchi, Hidetaka Yamaji, Shinnosuke Yamaoka, Toshihiko Uno, Mutsumi Watanabe, Taiji Sakamoto

**Affiliations:** 1 Department of Ophthalmology, Kagoshima University Graduate School of Medical and Dental Sciences, Kagoshima, Japan; 2 Shirai Eye Hospital, Kagawa, Japan; 3 Graduate School of Science and Engineering, Kagoshima University, Kagoshima, Japan

**Keywords:** Retinal diseases, Predictive markers

## Abstract

This study was to investigate the relationship between the metamorphopsia and foveal avascular zone (FAZ) parameter in eyes with epiratinal membrane (ERM). We studied patients with an ERM visited retinal service unit at the Kagoshima University Hospital or Shirai Hospital. The best-corrected visual acuity (BCVA), and the degree of metamorphopsia by M -CHARTS™ were evaluated. The 3 × 3 mm optical coherence tomography angiography (OCTA) images of the superficial layer were obtained. Area (mm^2^), the circularity, eigen value were calculated using ImageJ software. The relationship between visual function, such as best corrected visual acuity (BCVA) and metamorphopsia, and FAZ parameters were studied by Pearson’s correlational coefficient. Fifty-four eyes of 51 patients (24 men and 27 women) with an ERM were studied. The mean age of the patients was 69.6 ± 8.20 years. The mean BCVA and metamorphopsia score was 0.31 ± 0.29 logMAR units and 0.49 ± 0.42. There was no significant relationship between BCVA and FAZ parameters. While, metamorphopsia score was significantly and negatively correlated with all of FAZ parameters (area R = − 0.491, *P* < 0.001; circularity R = − 0.385, *P* = 0.004; eigenvalue ratio R = − 0.341; *P* = 0.012). Multiple regression analysis showed the FAZ area was solely and significantly correlated with metamorphopsia score (β − 0.479, *P* < 0.001). The size but not the shape of the FAZ was significantly correlated with the degree of metamorphopsia suggesting that it could be an objective parameter of metamorphopsia in ERM patients.

## Introduction

An epiretinal membrane (ERM) is a common retinal disorder that is identified as a translucent membrane on the surface of the retina^1–3^. The membrane can become thick and contractile, sometimes leading to impairment of the visual acuity and distortion of visual images^4^.

The surgery for ERM has been performed for years. However, its post-surgical recovery of metamorphopsia is not necessarily satisfactory. It was demonstrated that metamorphopsia was significantly associated with the quality of vision after ERM surgery^5,6^. Thus, assessing the severity of metamorphopsia and its change is crucial for deciding the surgery for ERM.

So far, Amsler grid chart has been used to evaluate metamorphopsia^7,8^. However, this cannot provide the quantitative value and is not always suitable for the changes after intervention. Recently, new method to semi-quantify the value of metamorphopsia is gaining interest such as M-CHARTS™^9^, Although this is superior to the previous method; the value is dependent upon the patient’s response which makes it difficult to interpret the result objectively.

To solve this problem, Okamoto et al. reported that the thickness of inner retina in optical coherence tomography (OCT) B scan image is significantly related to the degree of metamorphopsia^10^. Nonetheless, this method requires substantial amounts of measurement and complicated analysis. Therefore, an easy and objective method is needed.

OCT angiography (OCTA) is a new imaging technology that can obtain the images of the retinal vasculature non-invasively. The foveal avascular zone (FAZ) is a circular area with a diameter of 500 µm where the retinal capillaries are absent^11,12^. The clinical value of FAZ on OCTA has been extensively explored in many retinal diseases^11–15^. There are several reports on FAZ in ERM^16–19^. The area of FAZ has been found to become smaller according as the deterioration of ERM^16^. To our knowledge, however, the correlation between metamorphosis and FAZ has not been reported. In this study, we evaluated the relationship between visual function and FAZ parameter in eyes with ERM, paying special attention to metamorphopsia.

## Results

### Demographics of patients

The demographics of the patients are presented in Table 1. Fifty-four eyes of 51 patients, 24 men and 27 women with an ERM were studied. The mean age of the patients was 69.6 ± 8.20 years with a range of 47–87 years. The mean axial length was 23.9 ± 1.17 mm with a range of 21.85–26.66 mm. The mean best-corrected visual acuity (BCVA) was 0.31 ± 0.29 logarithm of the minimal angle of resolution (logMAR) units (Snellen visual acuity, 20/40.8). The mean horizontal and vertical metamorphopsia score was 0.50 ± 0.53 with a range of 0–2.0 and 0.48 ± 0.47 with a range of 0–2.0, respectively. The mean metamorphopsia score was 0.49 ± 0.42 with a range of 0–2.0. The mean central macular thickness (CMT) was 421 ± 78 μm with a range of 264–674 μm. (39 eyes of 37 patients).

**Table 1:** Demographics of patients.

	Mean ± SD	Range
Age	69.6 ± 8.2	47—87
Sex (Male / Female)	24/27	–
Axial length (mm)	23.9 ± 1.17	21.85–26.66
Visual acuity (logMAR)	0.31 ± 0.29	0–1.22
Metamorphopsia score (Horizontal)	0.50 ± 0.53	0–2.0
Metamorphopsia score (Vertical)	0.48 ± 0.47	0–2.0
Metamorphopsia score (Average)	0.49 ± 0.42	0–2.0

### Correlation between parameters of FAZ

The correlation between the parameters related to the size and shape of FAZ was examined for 27 eyes and 27 eyes. Supplementary Table S1 shows the FAZ parameters of the 27 eyes. Area, perimeter and Feret's diameter, which are parameters of the FAZ area, had a very strong correlation with a correlation coefficient of 0.94 or more. Similarly, circularity and solidity, parameters related to the shape of FAZ, had a strong correlation with a correlation coefficient of 0.893. The eigenvalue ratio, axial ratio and roundness had a perfect correlation with a correlation coefficient of − 1.0 or 1.0. Based on the above results, in this study, area, circularity, and eigen value were applied for the further analysis.

### Parameters of foveal avascular zone

Mean area of FAZ was 0.14 ± 0.08 mm^2^ with a range of 0.03–0.32. The mean circularity was 0.49 ± 0.11 with a range of 0.28–0.69. The mean eigen value was 0.65 ± 0.16 with a range of 0.27–0.96. The mean principal angle of direction was 32.8 ± 25.0 with a range of 0.1–88.4 (Table 2).

**Table 2:** Parameters of the foveal avascular zone.

	Mean ± SD	Range
Area (mm^2^)	0.14 ± 0.08	0.03–0.32
Circularity	0.49 ± 0.11	0.28–0.69
Eigen value	0.65 ± 0.16	0.27–0.96
The angle of the first principal component	32.8 ± 25.0	0.1–88.4

### Relationship between visual function and FAZ parameters

**Figure 1:**
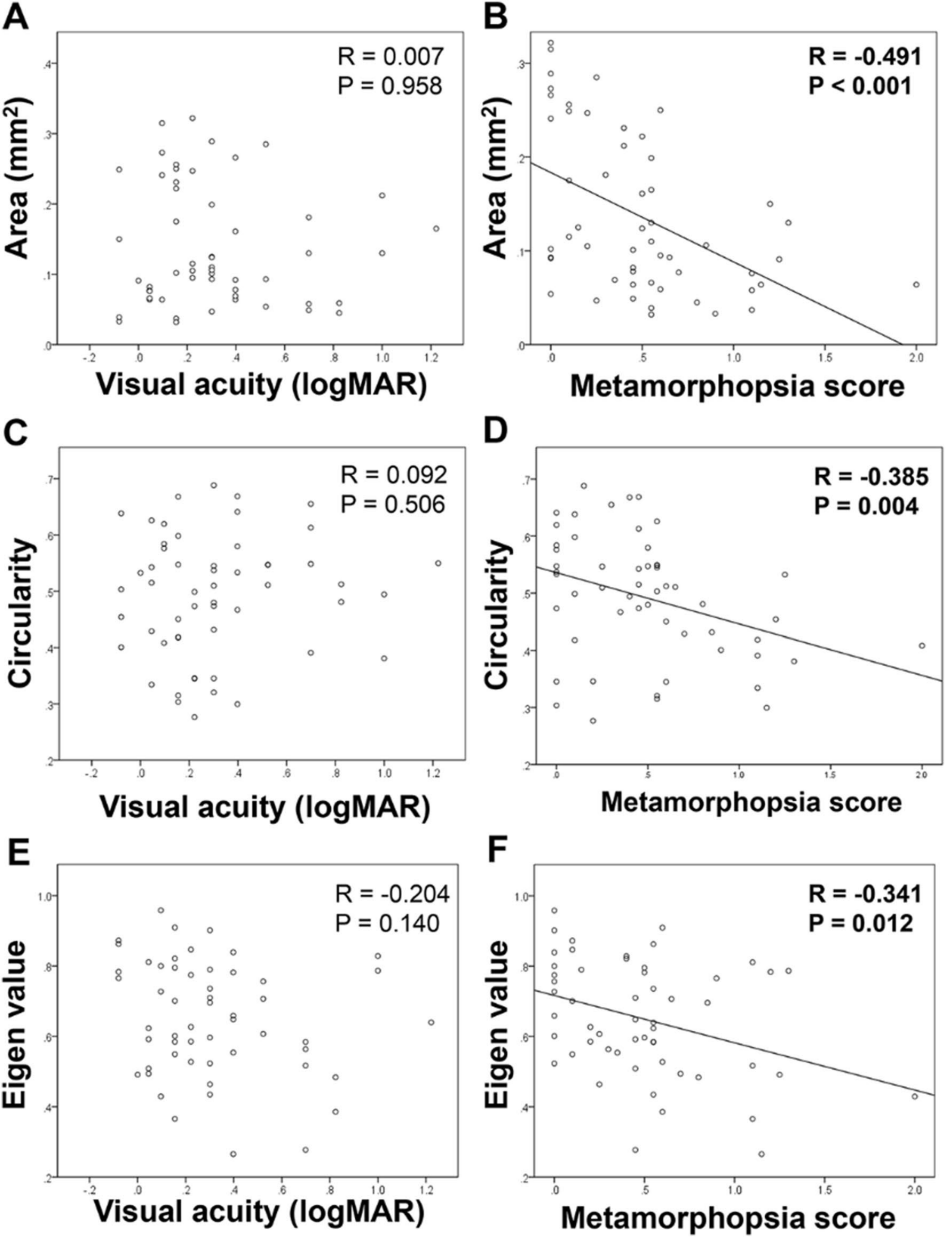
Relationship between visual function and FAZ parameters. There is no significant relationship between BCVA and FAZ parameters [(**A**) area R = 0.007, *P* = 0.958; (**C**) circularity R = 0.092, *P* = 0.506; (**E**) eigen value ratio R = − 0.204, *P* = 0.140]. While, metamorphopsia score is significantly and negatively correlated with all of FAZ parameters [(**B**) area R = − 0.491, *P* < 0.001; (**D**) circularity R = − 0.385, *P* = 0.004; (**F**) eigen value ratio R = − 0.341; *P* = 0.012].

There was no significant relationship between BCVA and FAZ parameters (Fig. 1A area R = 0.007, *P* = 0.958; 1C circularity R = 0.092, *P* = 0.506; 1E eigen value ratio R = − 0.204, *P* = 0.140). There was significant relationship between BCVA and CMT(R = 0.467, *P* < 0.001). While, metamorphopsia score was significantly and negatively correlated with all of FAZ parameters (Fig. 1B area R = − 0.491, *P* < 0.001; 1D circularity R = − 0.385, *P* = 0.004; 1F eigen value ratio R = − 0.341; *P* = 0.012). Multiple regression analysis showed the FAZ area was solely and significantly correlated with metamorphopsia score (β − 0.479, *P* < 0.001).

When the correlation between CMT and several other factors was examined in 39 eyes of 37 cases in which the CMT was measurable, a significant correlation was found between the CMT and the BCVA (R = 0.467, *P* = 0.003), and between the CMT and the degree of metamorphopsia (R = 0.475, *P* = 0.002). Multiple regression analysis with the FAZ parameters was performed, and a significant correlation was found in which only the CMT was independent of the BCVA (β = 0.467, *P* = 0.003). In addition, there was a significant independent correlation between the degree of metamorphopsia and the FAZ area (β = 0.524, *P* = 0.001).

### Comparison of visual function between the eyes with vertically- and horizontally-long FAZ

Among the subjects, 37 eyes (68.5%) have horizontally-long FAZ while 17 eyes (31.5%) have vertically-long FAZ (Table 3). Mean BCVA were 0.33 ± 0.27 and 0.28 ± 0.34 in horizontally- and vertically- long FAZ groups which was not significant between groups (*P* = 0.277). Mean metamorphopsia score were 0.45 ± 0.42 and 0.56 ± 0.44 in horizontally- and vertically-long FAZ groups which was not significant between groups (*P* = 0.415).

**Table 3:** Comparison of visual function between the eyes with vertically- and horizontally- long FAZ.

	Horizontally-long FAZ group	Vertically-long FAZ group	*P* value
Angle of the first principal component	0–45°	45–90°	
No. of cases	37 eyes	17 eyes	–
Visual acuity (logMAR)	0.33 ± 0.27	0.28 ± 0.34	0.277
Metamorphopsia score	0.45 ± 0.42	0.56 ± 0.44	0.415

### Correlation between parameters of FAZ and stage of ERM

**Figure 2:**
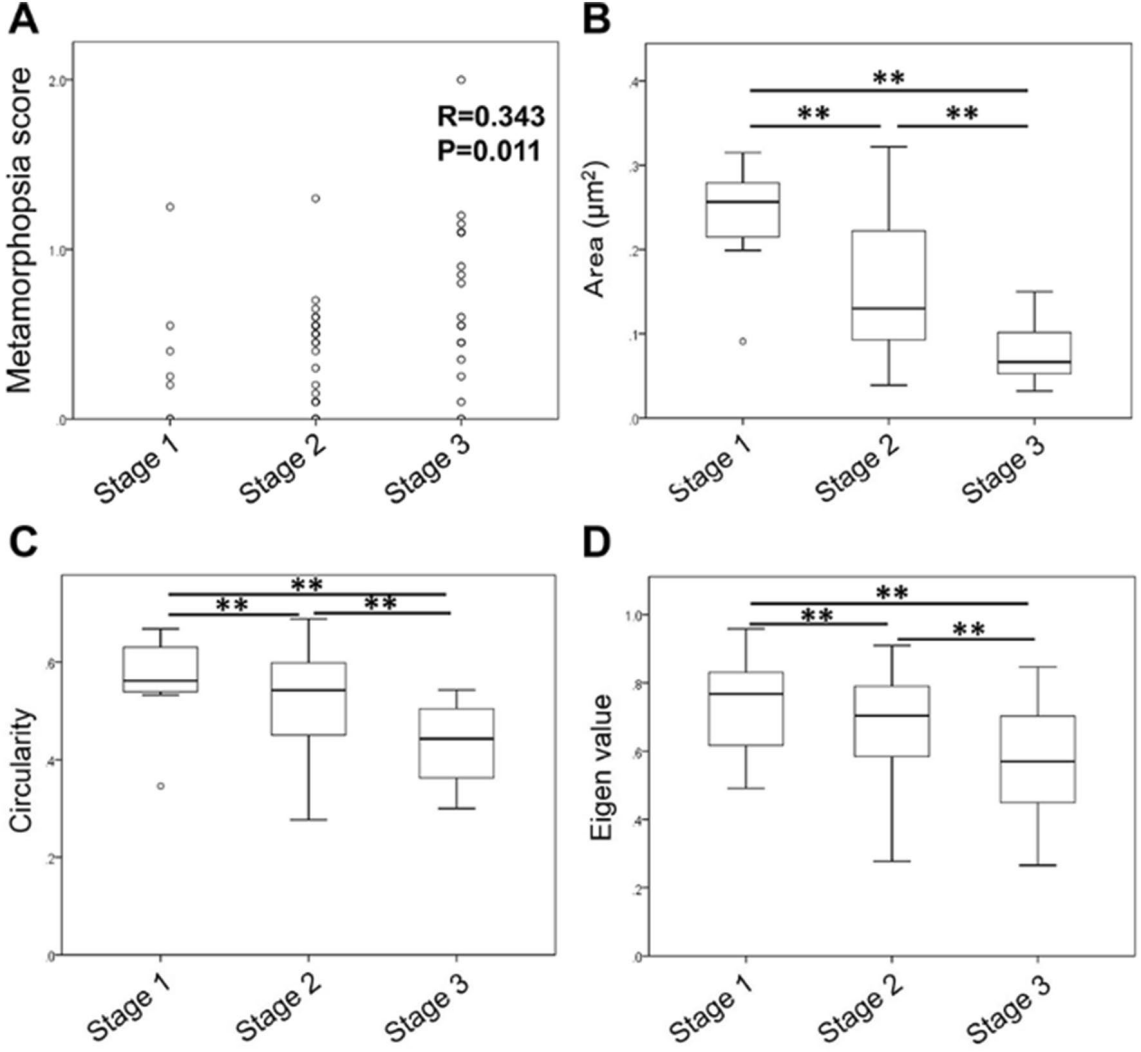
Correlation between parameters of FAZ and stage of ERM. Metamorphopsia score was significantly and positively correlated with the stage of ERM (**A**, R = 0.343, *P* = 0.011). Significant differences were observed among the three groups in all of the area (**B**, *P* < 0.01), circularity (**C**, *P* < 0.01), and eigenvalue ratio (**D**, *P* < 0.01).

Among the ERM eyes studied, 8 eyes were classified into stage 1, 26 eyes were stage 2, and 20 eyes were stage 3. No eye was classified into stage 4. Metamorphopsia score was significantly and positively correlated with the stage of ERM (Fig. 2A, R = 0.343, *P* = 0.011). Significant differences were observed among the three groups in all of the area (Fig. 2B, *P* < 0.001), circularity (Fig. 2C, *P* = 0.001), and eigenvalue ratio (Fig. 2D, *P* = 0.019). Table 4 shows the reduction rate of the parameters in each stages normarized by the score of stage 1. Although circulality and eigen value in stage 3 decreased with 22–23% compared to that in stage1, area in stage 3 decreased with 68% compared to that in stage1.

**Table 4:** FAZ parameters in different stages normalized by the score in ERM stage 1.

	Area	Circularity	Eigen value
Stage 1	1	1	1
Stage 2	0.64	0.93	0.93
Stage 3	0.32	0.77	0.78

## Discussion

**Figure 3:**
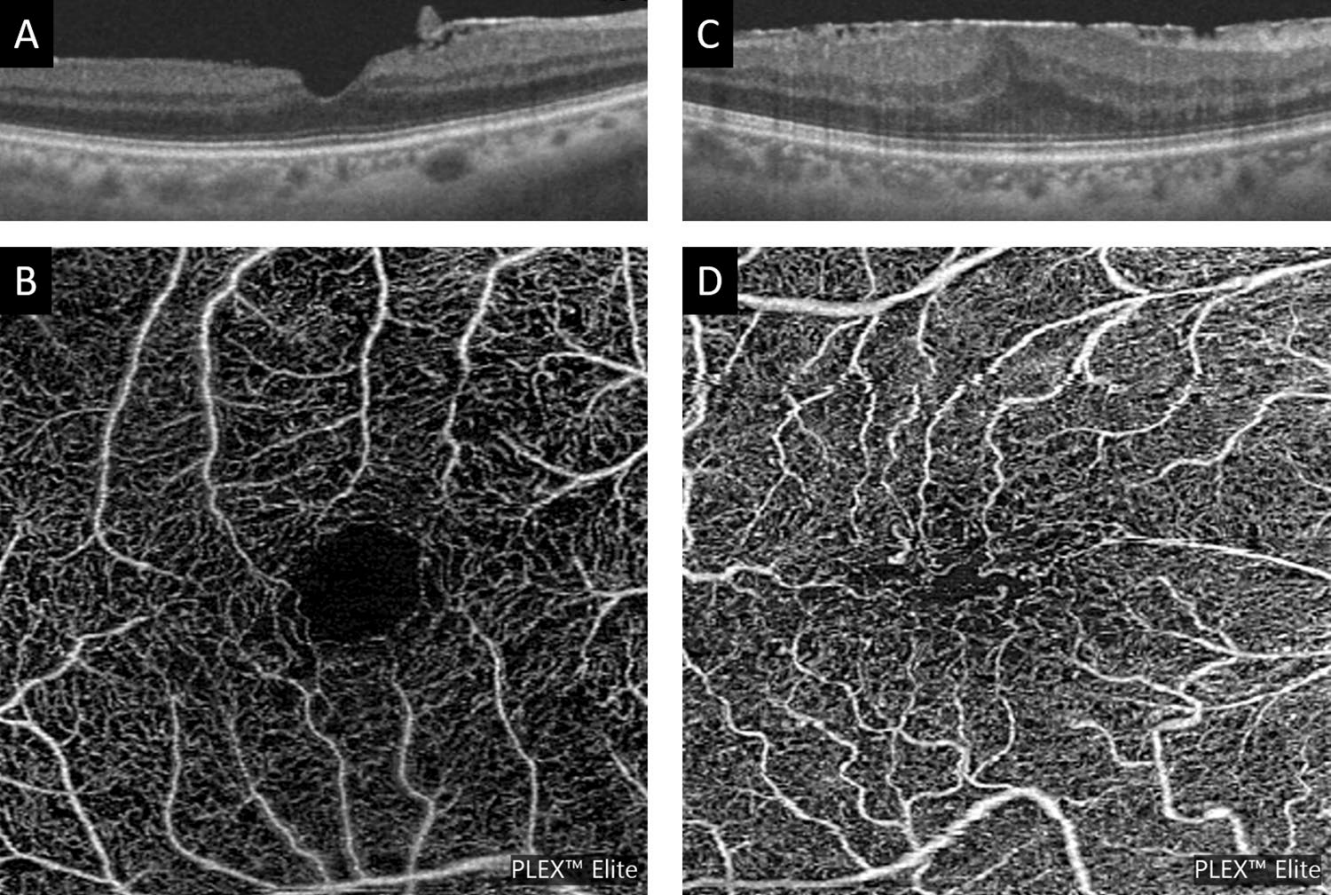
Representative cases showing differences in the FAZ area and shape. (**A**, **B**) A representative case of stage1 with metamorphopsia score of 0. The FAZ area is large and has a circular shape. (**C**, **D**) A representative case of stage3 with metamorphopsia score of 1.15. The FAZ area is small and has a horizontally-long FAZ.

In this study, we found that (1) the degree of metamorphopsia in ERM patients was significantly correlated with the area of the FAZ, (2) As the stage of ERM progresses, not only the FAZ area becomes smaller but also the shape becomes distorted (Fig. 3).

This is a reasonable result based on the process of ERM progression. ERM progresses from a transparent retinal membrane called the “cellophane macular reflex” that does not affect vision until becoming a thick fibrous membrane called the “preretinal macular fibrosis”^1–3^. When this fibrosis occurs on the macular area, the internal granular layer thickens and the foveal depression disappears. These changes deform the inner sensory retina, which contains the capillaries forming the FAZ, contracts. As a result FAZ becomes smaller in size. This observation was supported by previous results^16,17^.

Metamorphopsia is strongly associated with thicker INL^6,20^. Since the capillaries constituting FAZ are located in the retinal nerve fiber layer and ganglion cell layer which are in contact with INL, it is reasonable to correlate the area of FAZ with the degree of metamorphopsia. When the INL thickness was measured in 23 eyes by the same method as the previous report10, a significant correlation (R = − 0.743, *P* < 0.001, Spearman correlation coefficient) was found between the FAZ area and the INL thickness. In addition, there was a significant correlation (R = 0.744, *P* < 0.001) between the degree of metamorphopsia and INL thickness (see Supplemental FigureS1).

In ERM, small FAZ area and abnormal circularity/roundness of FAZ have been reported^19,21^. Although Govetto et al. mentioned that the FAZ became smaller according as the progression of stage of ERM, there was no supporting data^17^. We found that metamorphopsia increases and FAZ reduces in association with the progression of ERM. In the ERM Stage advocated by Govetto et al. foveal depression disappears from Stage 2, and the abnormality of the inner retinal layer is evident in Stage 3. The thickness of the foveal retina also increases with the stage^17^. Thus, the morphology of retinal nerve fiber layer and ganglion cell layer, which contain retinal capillaries, were changed from stage 2. As a result, the FAZ may become small and distorted with progression of ERM stage.

About 70% of shape of FAZ were horizontally long in ERM eyes. This means that the ERM contracts more vertically. The reasons for this can be anatomically explained as follows. First, the influence of the direction of retinal nerve fibers affects this phenomenon. The retinal nerve fibers from the optic nerve to the macula are arched and run horizontally^22^. When the ERM contracts in a horizontal direction, forces parallel to the nerve fiber running pattern are generated. Longitudinal contraction, on the other hand, applies vertical force to the nerve fiber. These forces certainly have different effects on the nerve fibers. Although there was no significant difference between horizontally- and vertically-shaped FAZs, these differences may influence the outcome. Second, the optic disc may be involved. When the ERM contracts on the macula, traction from the macula to the optic nerve is exerted only on the retinal nerve fibers leading to the optic nerve. That is, there are fewer “space” than other sites. This may result in less shrinkage in the horizontal direction. Finally, regional differences in the amount of nerve fibers may play a role. The amount of nerve fibers passing through the macula varies between region^23^. From the macula to the optic nerve, there are more nerve fibers than in other areas. This imbalance of retinal nerve fiber layer may also have influenced the results.

Each FAZ parameter was significantly correlated with the degree of metamorphopsia in the simple correlation analysis. However, multiple regression analysis showed that only the area of the FAZ was significantly correlated with the metamorphopsia. As the ERM stage progressed, all three FAZ parameters examined in this study decreased in value as the stage progressed. However, when looking at the rate of change, the circularity and the eigen value ratio are reduced by 22–23% between Stage 1 and 3, while the area is greatly reduced by 68% between them. The second possibility was that contractile force can work on every surface area of the retina in ERM. In that case, FAZ can get smaller without changing its shape. There were some cases with round shaped, but small FAZ in size (see Supplementary Figure S2).

On the other hand, the correlations between the FAZ parameters and the BCVA were not significant. Although the FAZ parameter is affected by the intensity of the ERM traction, the retinal capillaries that make up the FAZ are located within the outer nuclear layer of the retina and are away from the ellipsoid zone which directly affects the BCVA. The results indicate that the correlations among the FAZ parameters were weak. In a previous report10, there was a significant relationship between the BCVA and disruption of the ellipsoid zone in eyes with an epiretinal membrane, but the correlations between the retinal surface parameters and the BCVA were not significant.

There are limitations in this study. First, this is a retrospective study with limited number of ERM patients. Second, more than half of the cases were referred to university hospitals, so mild cases of ERM might not be included so many in this study. Third, we did not observe the recovery process of FAZ after surgery. Therefore it is not necessarily possible to say the size of FAZ can be a good indicator to monitor the metamorphopsia after surgery of ERM. Finally, the area of the FAZ differs between subjects such as sex, age. Thus, it is not suitable for comparison between individuals compared with the shape of FAZ^24^.

In conclusion, as ERM Stage progresses, the FAZ area becomes smaller and more distorted. The size of the FAZ was significantly correlated with the degree of metamorphopsia in ERM patients. The size of FAZ is useful for the follow up of the metamorphopsia of the ERM patient.

## Methods

### Ethics statement

This was a retrospective cross-sectional study performed at Kagoshima University Hospital, Kagoshima, Japan and Shirai Hospital, Kagawa, Japan. A written informed consent was obtained from all the subjects after an explanation of the procedures to be used and possible complications. The procedures used were approved by the Institutional Review Board of Kagoshima University hospital, and they conformed to the tenets of the 1989 Declaration of Helsinki.

### Subjects and examination methods

Patients with an ERM visited retinal service unit at the Kagoshima University Hospital or Shirai Hospital from January 2017 to August 2017 were studied. The exclusion criteria were; eyes with high myopia (< − 6 diopter), eyes with opacity of the transparent media which affects the image quality of OCTA, eyes with indistinct or unclear margins of the FAZ, and eyes with other diseases such as DR and glaucoma. The decision to exclude an eye was made by two independent raters (HS, HT). Eyes were excluded if either of the rater determined that it had met at least one of the exclusion criteria. All of the eyes had a comprehensive ocular examination which included slit-lamp examinations of the anterior segment of the eye and ophthalmoscopic examinations of the fundus. The intraocular pressure was measured with a pneumotonometer (CT-80, Topcon, Tokyo, Japan), and the axial length was measured with the AL-2000 ultrasound instrument (Tomey, Tokyo, Japan). The BCVA was measured after determining the refractive error with an Auto Kerato-Refractometer (RM8900, Topcon). The degree of metamorphopsia was evaluated by M-CHARTS (Inami Co., Tokyo, Japan). M-CHARTS examination collects the metamorphopsia score of vertical line and horizontal line. We use the average score of vertical and holizontal lines as reported^5,6,10^.

### Imaging protocol

OCTA images were obtained using a swept-source OCT device (Plex Elite 9000, Carl Zeiss, San Leandro, CA) with a central wavelength of 1040–1060 nm, an acquisition speed of 100,000 A-scans/sec, and an axial and transversal resolution of 5 and 14 μm in tissue, respectively. Scans were taken from 3 × 3 mm cubes with each cube consisting of 300 clusters of four repeated B-scans centered on the fovea. To determine the CMT, 7 × 7 mm cube images were obtained centered on the fovea by another swept-source OCT (DRI OCT Triton; Topcon, Tokyo, Japan). A whole retinal thickness map centered on the fovea was created using the Early Treatment Diabetic Retinopathy Study grid. The CMT was calculated by averaging the retinal thickness of the macula within 1 mm from the fovea.

### Analysis of area and shape of foveal avascular zone

The OCTA images of full thickness slab were obtained as 1024 × 1024 pixel tagged image file format (TIFF) images. It was analyzed by ImageJ software to calculate the FAZ (ImageJ version 1.47, National Institutes of Health, Bethesda, MD; available at: https://imagej.nih.gov/ij/).

We used OCTA images of full thickness slab because that of deep layer can be affected by projection artifact^25^. Although some of research about FAZ of OCTA images in ERM cases used the images of superficial layer^18,19,25–27^, our preliminary analysis and previous work showed the capillary images between OCTA images of full thickness slab and superficial layer are comparable^28^.

The FAZ area was defined as the avascular area in the center of the fovea, and the border of the FAZ was manually drawn by a single retina specialist (HS) who were masked to the clinical information.

Area (mm^2^), perimeter, feret’s diameter as the parameter for FAZ size and the circularity, solidity, roundness, eigen value and axial ratio as the parameter for FAZ shape were calculated using ImageJ as reported^24^. The area and perimeter length were measured using the ImageJ software. Feret’s maximum diameter of the FAZ, also known as the maximum caliper, was measured with the ImageJ software.

The circularity is a shape descriptor that can mathematically indicate the degree of similarity of the FAZ to a perfect circle. A value of 1.0 designates a perfect circle, and as the circularity value decreases, the shape is increasingly less circular.

$$ {\text{Circularity is defined by the equation: Circularity }} = 4\pi \times {\text{area}}/{\text{perimeter}}^{2} $$

The roundness uses the best fit ellipse, and is similar to circularity but is not sensitive to irregular borders along the perimeter of the FAZ. Roundness is defined by the equation:

$$ {\text{Roundness}} = 4\pi \times {\text{area}}/\left( {\text{length of major axis}} \right)^{2} $$

The axial ratio is obtained from a best fit ellipse of the FAZ. The following parameters were determined from the best fit ellipse: the length of the major and minor axes and the axial ratio. The axial ratio is calculated by the following equation:

$$ {\text{Axial ratio}} = \left( {\text{length of major axis}} \right)/\left( {\text{length of minor axis}} \right) $$

Solidity describes the extent to which a shape is convex or concave. The area enclosed by a convex hull can provide information regarding the solidity of the shape. The solidity of a completely convex shape is 1, the farther the solidity deviates from 1, the greater the extent of concavity in the structure.

$$ {\text{Solidity is defined by the equation:}}\;{\text{Solidity}} = {\text{area}}/\left( {\text{convex area}} \right) $$

The eigen value is the outline of FAZ was expressed on the point coordinates, and the average value of all points is set as the origin (0). Next, standardization is performed so that the average of each coordinate is 0 and the variance is 1. The major axis of the ellipse of the standardized data was defined as the first principal component and the minor radius was defined as the second principal component, and the ratio was defined as the eigen value ratio.

### Correlation between each parameter

There are many parameters related to the size and shape of the FAZ, but the correlation between them is very high in some of them^24^. Therefore, the correlation of the FAZ parameters was examined in 27 eyes in order to avoid duplication of the FAZ parameters having a very high correlation.

### Comparison of FAZ parameter and visual function

The relationship between visual function, such as visual acuity and metamorphopsia (metamorphopsia score, an average of horizontal and vertical scores by M-CHARTS 5,6,10), and FAZ parameters, such as area, circularity, and eigen value, were studied. Furthermore, multiple regression analysis was performed to eliminate confounding among FAZ parameters.

### Comparison of vertically- and horizontally-long FAZ

In preliminary experiment, we realized that there were different shapes of FAZs, vertically or horizontally long-shaped FAZ. We decided the FAZ in which the principal angle of direction was between 0° and 45° as horizontal long FAZ and that in which the principal angle of direction was from 45° to 90° as vertically long FAZ. We compared the visual function between the groups.

### Correlation between the stage of ERM and FAZ parameters

The stage of ERM in each eye was determined by two masked examiners (HS and HT) based upon the OCT B scan image in earlier report^17^. The correlation between ERM stage and metamorphopsia score or FAZ parameters were analyzed.

### Statistical analyses

All statistical analyses were performed with SPSS statistics 19 for Windows (SPSS Inc., IBM, Somers, New York, USA). The coefficient of variation (SD/mean) also was calculated. Relationship between the BCVA and metamorphopsia score and FAZ parameter were analyzed using Pearson’s correlational coefficient. A stepwise forward multivariate linear regression analysis was performed to evaluate the contribution of clinical findings to each morphologic parameter. The correlation between the stage of ERM and each biomarker was done by Kruskal–Wallis test. A *P* value of 0.05 was considered to be statistically significant.

## Supplementary information

Supplementary file1
