# Supplementary material for: Association of foveal avascular zone with the metamorphopsia in epiretinal membrane
Source: Sci Rep. 2020 Oct 13;10:17092. doi: 10.1038/s41598-020-74190-x (PMC7555497; doi:10.1038/s41598-020-74190-x)
Supplement: Supplementary file 1 — Supplementary file1 [file 41598_2020_74190_MOESM1_ESM.docx]

**Association of foveal avascular zone with the metamorphopsia**

**in epiretinal membrane**

Hideki Shiihara^1^, Hiroto Terasaki^1^, Shozo Sonoda^1^, Naoko Kakiuchi^1^, Hidetaka Yamaji^2^, Shinnosuke Yamaoka^3^, Toshihiko Uno^2^, Mutsumi Watanabe^3^, Taiji Sakamoto^1^

1. Department of Ophthalmology, Kagoshima University Graduate School of Medical and Dental Sciences, Kagoshima, Japan

2. Shirai eye hospital, Kagawa, Japan

3. Graduate School of Science and Engineering, Kagoshima University, Kagoshima, Japan

**Supplementary Table S1. Correlation between parameters of FAZ**

|  | Perimeter | Feret’s diameter | Circularity | Solidity | Eigen value | Axial ratio | Roundness |
| --- | --- | --- | --- | --- | --- | --- | --- |
| Area | R=0.955  P<0.001 | R=0.943  P<0.001 | R=0.554  P=0.003 | R=0.470  P=0.013 | R=0.410  P=0.034 | R=-0.410  P=0.034 | R=0.410  P=0.034 |
| Perimeter | - | R=0.964  P<0.001 | R=0.319  P=0.105 | R=0.254  P=0.202 | R=0.648  P<0.001 | R=-0.648  P<0.001 | R=0.648  P<0.001 |
| Feret’s diameter | - | - | R=0.356  P=0.068 | R=0.322  P=0.102 | R=0.538  P=0.004 | R=-0.538  P=0.004 | R=0.538  P=0.004 |
| Circularity | - | - | - | R=0.893  P<0.001 | R=0.419  P=0.029 | R=-0.419  P=0.029 | R=0.419  P=0.029 |
| Solidity | - | - | - | - | R=0.208  P=0.299 | R=-0.208  P=0.299 | R=0.208  P=0.299 |
| Eigen value | - | - | - | - | - | R=-1.000  P<0.001 | R=1.000  P<0.001 |
| Axial ratio | - | - | - | - | - | - | R=-1.000  P<0.001 |

**Supplementary Figure S1. Relationship inner nuclear layer thickness and area of FAZ and metamorphopsia**

**
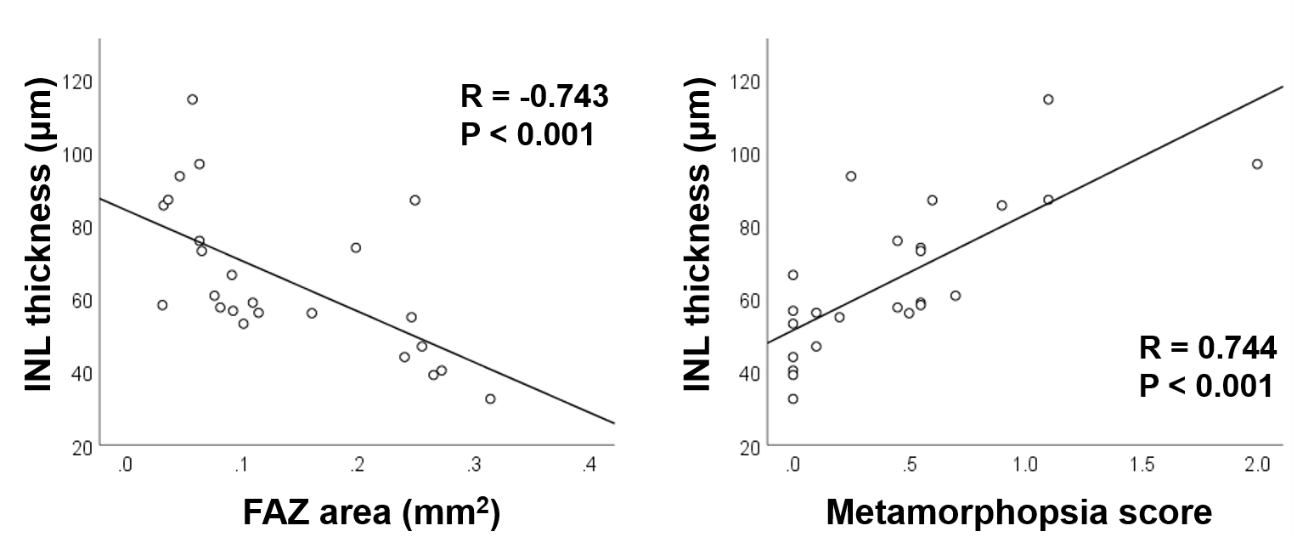
**

**Supplementary Figure S2. Representative case with round shaped, but small FAZ in size.**


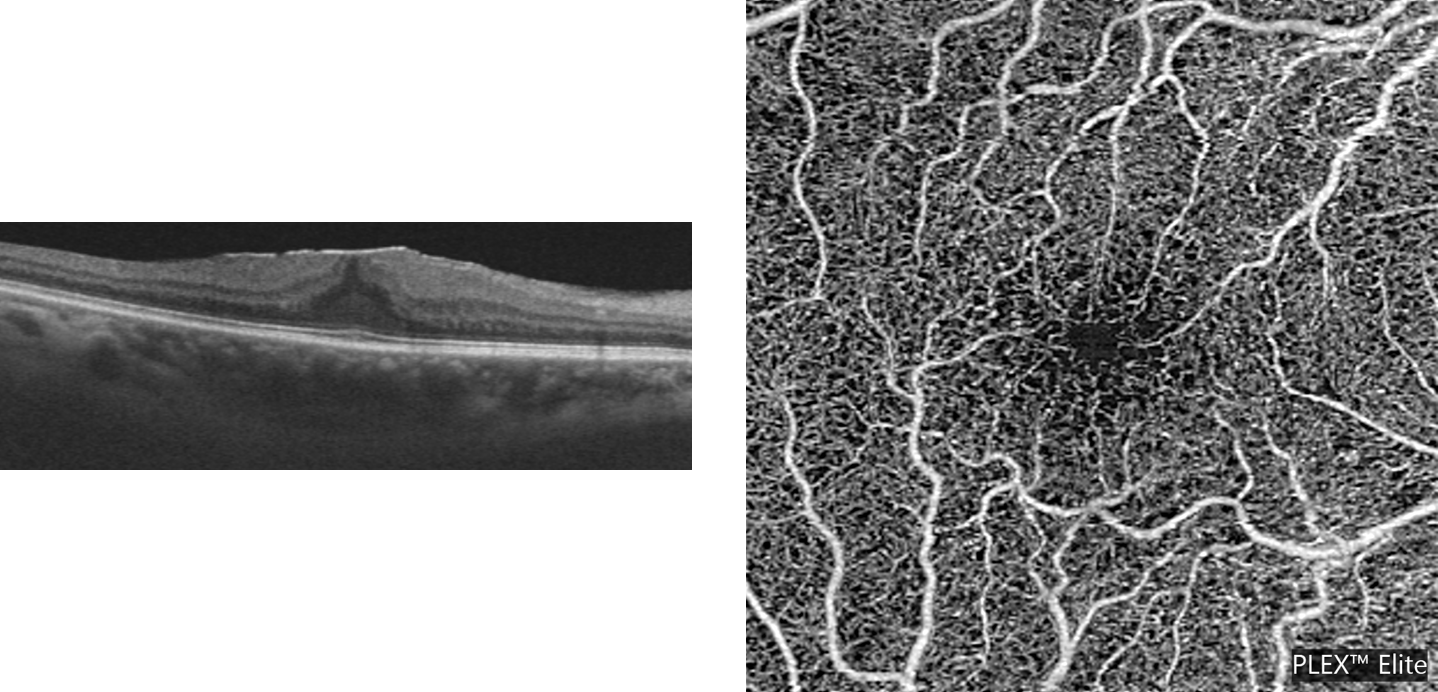


The area of the FAZ was 0.039 mm2, the circularity was 0.50, and the eigen value was 0.86. The area of FAZ was small and the metamorphopsia was relatively strong, but the shape of FAZ was circular.
